# Seroconversion, genotyping, and potential mosquito vector identification of Japanese encephalitis virus in pig sentinel settings in Bali, Indonesia

**DOI:** 10.14202/vetworld.2024.89-98

**Published:** 2024-01-08

**Authors:** I Made Kardena, Anak Agung Ayu Mirah Adi, I Nyoman Mantik Astawa, Ida Bagus Made Oka, Shafi Sahibzada, Mieghan Bruce, Mark O’Dea

**Affiliations:** 1 Department of Pathobiology, Faculty of Veterinary Medicine, Udayana University, Jalan PB Sudirman, Denpasar, Bali, 80234, Indonesia; 2 School of Veterinary Medicine and Centre for Biosecurity and One Health, Harry Butler Institute, Murdoch University, Perth, Western Australia, 6150, Australia; 3 Australian Centre for Disease Preparedness, Commonwealth Scientific and Industrial Research Organization, Geelong, VIC 3220, Australia

**Keywords:** genotyping, Japanese encephalitis virus, pig sentinel setting, potential mosquito vectors, seroconversion

## Abstract

**Background and Aims:**

Despite the endemicity of Japanese encephalitis virus (JEV) in humans and animals in the Province of Bali, Indonesia, there is little data on whether seroconversion to the virus occurs in pigs, JEV genotypes circulating, and it’s potential mosquito vectors in the area. The aims of this study were to (i) Determine whether JEV infection in Balinese pigs occurs before reaching their sexual maturity, (ii) identify the genotypes of circulating JEV, and (iii) identify potential JEV mosquito vectors at the study sites in urban and peri-urban areas of Bali.

**Materials and Methods:**

Sixteen 1-week-old Landrace piglets from two different sows were housed in Denpasar. Similarly, 18 one-week-old mixed-breed piglets of two different sows were housed in Badung Regency. The piglets were bled every 1 to 4 weeks for up to 24 weeks. Serum samples from the 11 piglets were tested for antibodies against JEV, and seroconversion-suspected sera were titrated using an enzyme-linked immunosorbent assay. Blood of seroconverted sera from pigs were tested using polymerase chain reaction (PCR) to detect the genetic sequence of JEV. The mosquitoes in the sentinels were trapped throughout the study period to identify the potential mosquito vectors of JEV.

**Results:**

Antibodies were detected in most of the selected piglets’ sera from weeks 1 to 24 of their age. However, sera of pig B9 collected from the sentinel setting in Badung Regency showed a four-fold increase in antibody titer from week 4 to week 8, indicating seroconversion. PCR testing of blood from B9 (pooled blood sample collected from week 5 to week 8) identified JEV nucleic acids, which were phylogenetically classified as belonging to the JEV genotype III. Meanwhile, 1271 of two genera of mosquitoes, *Anopheles* spp. and *Culex* spp. were trapped in the pig sentinels.

**Conclusion:**

JEV seroconversion likely occurs before the pig reaches sexual maturity in Badung Regency. Sequence data indicate that JEV genotype III is circulating in the pig sentinel setting in the regency; however, circulating genotypes need to be clarified through increased surveillance. Meanwhile, *Culex* spp. and most likely *Culex quinquefasciatus* and *Anopheles* spp. were the dominant mosquitoes present in the study sites set in the urban area of Denpasar and peri-urban areas of Badung, Bali, indicating that these are likely vectors in spread of JEV in the region.

## Introduction

Japanese encephalitis (JE) is a zoonotic viral disease that is particularly prevalent in rural areas of Southeast Asia due to the close association of reservoirs and amplifying hosts in pig farms and rice paddy fields [1]. An increase in JE cases in urban and peri-urban areas has recently been reported in Asian countries, including Cambodia [2], Vietnam [3], Lao PDR [4], India [5], and Indonesia [6]. In the Indonesian Province of Bali, human JE cases have been reported to be the highest nationally [6] and occur in rural, urban, and peri-urban areas [7, 8].

The high demand for pigs and their related products has contributed to the high density of pig populations in Bali due to Balinese sociocultural factors. It is hypothesized that the high circulation and transmission of the JE virus (JEV) in the province is due to the presence of pigs, a known amplifying host [9]. In addition, mosquitoes that feed on viremic animals are responsible for the transmission of the virus to other susceptible hosts, including humans. *Culex* spp., primarily *Culex tritaeniorhynchus* [10], is the main mosquito vector. This vector has been reported to be the dominant mosquito host in rural areas [11]. Although *Aedes* spp., *Anopheles* spp., and *Mansonia* spp. have been reported to be potential vectors of the virus [12], limited studies are available on the potential mosquito vectors related to JEV transmission in urban and peri-urban areas. Therefore, to determine which mosquito populations contribute to JEV transmission, assessing potential mosquito vectors in the areas where human JE cases are detected is important. In addition, although four of the five genotypes (genotypes I to IV) have been documented in Indonesia [13], despite the endemicity of JEV in humans and animals in Bali [14], there is little data provided on which JEV genotypes are circulating in the area. To better understand the existence and circulation of the virus circulating in Bali compared to other areas in the country, studies on genotyping the virus circulating in Bali are required.

Although JEV is a significant public health priority, outbreaks of JEV can result in production economic loss for pig farmers, especially when the infection in susceptible pigs occurs after sexual maturity [15]. Infection during early gestation in naïve sows may result in abortion. In late pregnancy, infection leads to mummification or stillbirth, followed by infertility [2]. In newborn piglets, the infection may result in fever, depression, and hind limb tremors [16]. However, post-exposure antibodies can protect sows from reproductive disorders in subsequent seasons [17].

To date, no longitudinal studies in different geographical settings have been conducted to determine the age at which pigs seroconvert to JEV in endemic areas of Bali. To predict the period of the infection in a natural setting, a seroconversion assessment was performed in a sentinel study to estimate the occurrence of natural JEV infection in pigs in Bali. In addition, the identification of circulating JEV genotypes and the potential mosquito vectors that contribute to JEV transmission in Bali are needed to model dynamics of JEV in the region.

The aims of this study were as follows: (i) To estimate whether JEV infection in Balinese pigs occurs before reaching their sexual maturity; (ii) to identify the genotypes of JEV circulating at the study sites; and (iii) to identify the dominant JEV potential mosquito vector at the study sites that were set in urban and peri-urban areas of Bali.

## Materials and Methods

### Ethical approval

The study was approved by the Animal Ethics Committee of Murdoch University, Australia, with protocol ID 703, Permit number R3207/19, and Ethics Commission for the Use of Animals in Research and Education, Faculty of Veterinary Medicine, Udayana University, with reference number 15/UN.14.2.9/PD/2020.

### Study period and location

The study was conducted from September 2020 to February 2021 in the Province of Bali, Indonesia.

### Study design and sampling

**Figure-1:**
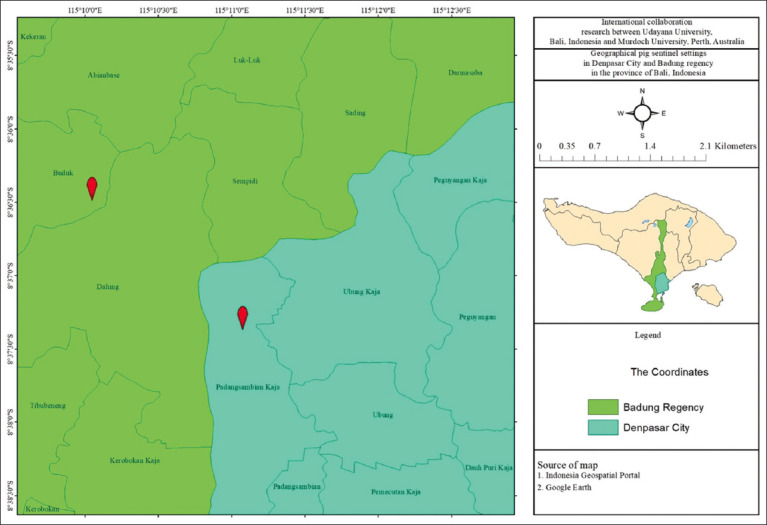
Geographical pig sentinel settings located in Denpasar and Badung, Bali Province.

Thirty-four piglets (7 days old) of four parity-three farrowing sows were used in two different sentinel settings in urban and peri-urban areas of Bali (Figure-1). A total of 16 Landrace piglets from two different sows were used in a 120 m^2^ pen located in Denpasar (geographical coordinate −8.6209; 115.1843). Meanwhile, the other 18 mixed-breed piglets from two different sows were also used and placed in a 100 m^2^ pen in Badung Regency (geographical coordinate −8.6057; 115.1673) (Table-1). All sampled piglets were born on site and were weaned on days 28–30. The sows and the piglets were reared in smallholder- or household-level pig farms in the study sites.

**Table-1:** Four sows and 34 piglets were used in this study that set in Denpasar and Badung, Bali Province.

Sows	Number of piglets	

	Female	Male
Sow Denpasar 1	4	2
Sow Denpasar 2	5	5
Sow Badung 1	5	3
Sow Badung 2	6	4

### Pig blood collection

Three milliliters of blood was collected from each pig via the jugular vein using sterile disposable 5 mL syringes with 23–22 G needles (depending on the age or weight of the pigs). The collected blood was divided into two parts: 1.5 mL of the blood was clotted for serum collection [poured in a plain coded VacuLab (Sanli Medical, China) glass tube], and the remaining blood was placed in a plain coded ethylenediaminetetraacetic acid-treated VacuLab (Sanli Medical) glass tube and stored at −70°C for polymerase chain reaction (PCR). The sows farrowing the sampled piglets were also bled a week after farrowing (at the same time as the first blood collection of the sampled piglets) to detect JEV antibodies. The clinical signs of all sample piglets were monitored during the study period.

### Pig serum sample analysis

Samples were screened for the presence of JEV antibodies using a commercial immunoglobulin G (IgG) enzyme-linked immunosorbent assay (ELISA) kit (https://www.elabscience.com/Manual/elisa_kits/E-AD-E002.pdf) according to the manufacturer’s instructions. Five (D2, D4, D6, D12, and D14) out of 16 pig sera collected from the sentinel in Denpasar and six (B3, B7, B9, B13, B15, and B17) out of 18 pig sera collected from the sentinel in Badung were subjected to the ELISA. Sample to positive ratios were calculated for serum samples to screen for evidence of an increase in IgG indicating seroconversion and compared across time points. Serum samples suspected to demonstrate seroconversion were serially titrated to determine whether there was a significant increase in JEV antibodies.

### Titration of suspected seroconverted sera

Titration was performed using an ELISA kit for porcine antibodies against JEV (Elabscience, China). In the titration, seroconversion-suspected pig serum samples showing seroconversion in the screening test were initially diluted to 1:40 and then serially diluted two-fold using the sample diluent provided in the kit. Antibody titers in the titrated serum samples were calculated on the basis of the optical density (OD) values measured in the sera that exceeded the kit cutoff of 0.2. Titers are expressed as the reciprocal value of the final dilution, demonstrating a positive reaction.

### Mosquito trap and identification

Mosquito light traps have been used to trap mosquitoes around the pig sentinels. Two unbaited outdoor 360–400 nm ultraviolet (UV) light traps (Krisbow^®^, China) were positioned in the sentinel pig farms a day before collecting the blood of the sentinel pigs. Light traps were installed, and the UV lamps in the traps were turned on at 6 pm for 12 h. Trapped mosquitoes were placed in an ice box with dry ice and transported to a laboratory for identification.

Identification of the trapped mosquitoes was based on pictorial identification key of important disease vectors of the World Health Organization (WHO) Southeast Asia Region [18] using a stereo microscope (Carl Zeiss Microlmaging GmbH, Germany). *Culex* spp. and all other mosquitoes were identified at the species level and genus level, respectively.

### Ribonucleic acid (RNA) extraction, primers, reverse transcription-PCR (RT-PCR), and sequencing

RNA extraction was performed using TRIzol LS (Invitrogen, USA) according to the manufacturer’s instructions.

Two pairs of primers were used to run the RT-PCR, JEV pre-membrane capsid gen fwd 5’-GTG GCT CGC AAG CTT GGC AG-3’ and rev 5’- CCA CGT CCT CTG GAT CAT TGC -3’ and JEV envelope (E) fwd 5’- AGT TAA CAT CAG GCC ACC TGA -3’ and rev 5’- GTT CCA TCT CGA CCA GCA C -3’ (OIE Terrestrial Manual 2018 online access: https://tinyurl.com/mv7s45ua.

All samples were tested using both primer pairs. Sequencing was performed using primer E. Bands of the expected size were excised and sent to PT Genetika Science Indonesia, Tangerang for Sanger sequencing.

A live attenuated JEV vaccine (14-14-2 JE; Chengdu Institute of Biological Products) was used as a positive control in the RT-PCR test performed in this study.

Total extracted RNA was reverse transcribed into complementary deoxyribonucleic acid (cDNA) using superscript III (Invitrogen). The cDNA was then amplified by PCR with 10 mM of each of the fwd and rev primers (IDT, Singapore). Reverse transcription was performed at 50°C for 1 h, followed by initial inactivation at 95°C for 7 min, and amplification (denaturation at 94°C for 30 s, annealing at 50°C for 30 s, and extension at 72°C for 90 s) was repeated 34 times in a thermocycler (MJ Mini, Bio-Rad Laboratories Inc., USA). The products were electrophoresed in a 1% agarose gel stained with ethidium bromide and visualized under UV light.

### Phylogenetic analysis

Sequencing results were analyzed by manually aligning the trimmed gene sequences with other related taxa of the JEV E gene using Clustal W (https://www.megasoftware.net/). Maximum likelihood phylogeny was performed using the Kimura two-parameter model and a maximum likelihood tree constructed with 1000 bootstrap replicants using the molecular evolutionary genetics analysis (MEGA) X software (https://www.megasoftware.net/).

## Results

### Antibodies against JEV detection and titration

Based on the ELISA test performed in the piglet blood samples of D2, D4, D6, D12, D14, B3, B7, B9, B11, B13, and B17 collected in week 1 to week 8, all of the OD values of ELISA test showed over the cutoff ranging from 0.6 to more than 2.2. JEV antibodies in the four sows of the piglet samples were also positively detected (Appendix 1). However, weekly changes in the ELISA OD values in the tested sera collected from week 1 to week 8 showed increased values of D2 from week 1, week 2, and week 4; D12 from week 1, week 3, and week 5; and B9 from week 1, week 4, and week 8, indicating seroconversion. The samples were chosen to be titrated to determine the JEV antibody titers.

The first titration was conducted on the sera of D2 collected in weeks 1, 2, and 4; the sera of D12 collected in weeks 1, 3, and 5; and the sera of B9 collected in weeks 1, 4, and 8 using ELISA OD values (Table-2).

**Table-2:** OD values titration of pig serum sample D2 in week 1, 2, and 4; D12 in week 1, 3, and 5; and B9 in week 1, 4, and 8.

Sample ID	D2			D12			B9			Blank wells	Blank wells	+ve and -ve control wells

	W1	W2	W4	W1	W3	W5	W1	W4	W8			

	1	2	3	4	5	6	7	8	9	10	11	12
A	2.474	1.5	1.926	1.819	1.127	2.057	1.761	0.92	1.71	0.228	0.419	2.901
B	1.695	1.046	1.445	1.42	0.862	1.578	1.332	0.55	0.905	0.139	0.517	2.846
C	1.286	0.714	0.909	1.144	0.618	1.084	0.944	0.306	0.663	0.119	0.615	2.897
D	1.049	0.524	0.596	0.728	0.361	0.646	0.548	0.186	0.418	0.156	0.566	0.068
E	0.759	0.31	0.376	0.582	0.213	0.407	0.343	0.133	0.286	0.166	0.347	0.065
F	0.531	0.202	0.215	0.341	0.155	0.214	0.193	0.092	0.155	0.186	0.351	0.07
G	0.344	0.155	0.148	0.178	0.121	0.147	0.125	0.079	0.107	0.161	0.299	0.06
H	0.21	0.104	0.101	0.112	0.083	0.093	0.095	0.066	0.085	0.176	0.179	0.061

*OD values obtained from 10A to 10H, 11A to 11H, 12G and 12H were from blank wells, while the values from 12A, 12B, and 12C wells were the positive controls; 12D, 12E and 12F were the negative controls. OD=Optical density, W=Week

The titration results of the D2 sera collected at weeks 1, 2, and 4 showed no indication of seroconversion. In week 1, the antibody titer was 5120, whereas in week 2, the titer decreased to 1280. It was stable at the same titer until week 4, when no seroconversion occurred.

Similarly, in the D12 titration sera, the serum at week 1 had an antibody titer of 2560, whereas at week 3, the titer decreased to 640, and at week 5, the titer increased to 1280. The two-fold increase in antibody titer from week 3 to week 5 was not significant and it was not conclusive that this change was due to seroconversion.

However, in the 1^st^ week of serum collection, B9 had a titer of 1280. The titer notably dropped to 320 at week 4 of serum collection and then significantly increased four-fold to 1280 at week 8. This significant increase indicates that seroconversion occurred between week 4 and week 8.

**Figure-2:**
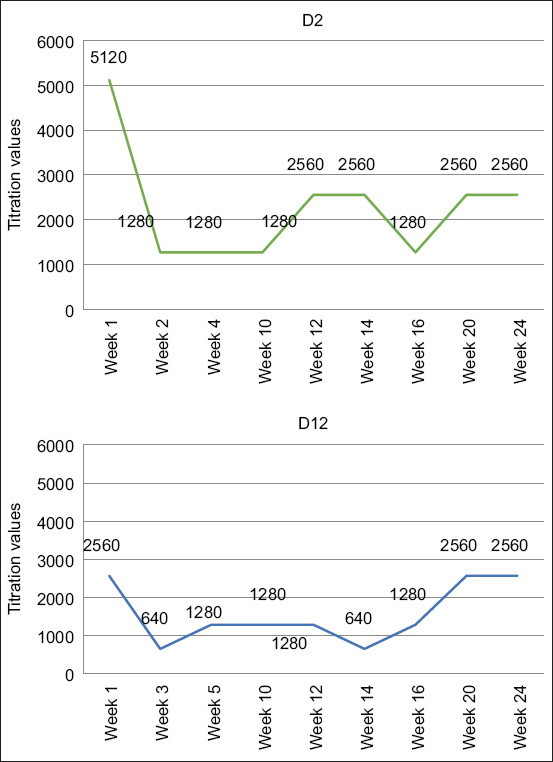
Titration of the pig serum samples D2 and D12 collected from week 1 to week 24.

Using the same method, serum samples of D2 and D12 collected at weeks 10, 12, 14, 16, 20, and 24 were then titrated. However, no seroconversion was observed on the basis of the ELISA OD values. Figure-2 presents the titration results of D2 and D12 pig serum samples collected from weeks 1 to 24. No classical pattern consistent with seroconversion was observed in these titration results. However, all of the titration results showed that JEV antibodies were detected in all periods of serum collection without a significant increase observed between weeks 1 and 24 of pigs’ age.

### Genotyping and PCR of JEV from the pig blood samples

Bands of the expected size were amplified from B9 blood samples A5595, a pooled blood sample collected from week 1 to week 4, and A5592, a pooled blood sample collected from week 5 to week 8 were identified.

**Figure-3:**
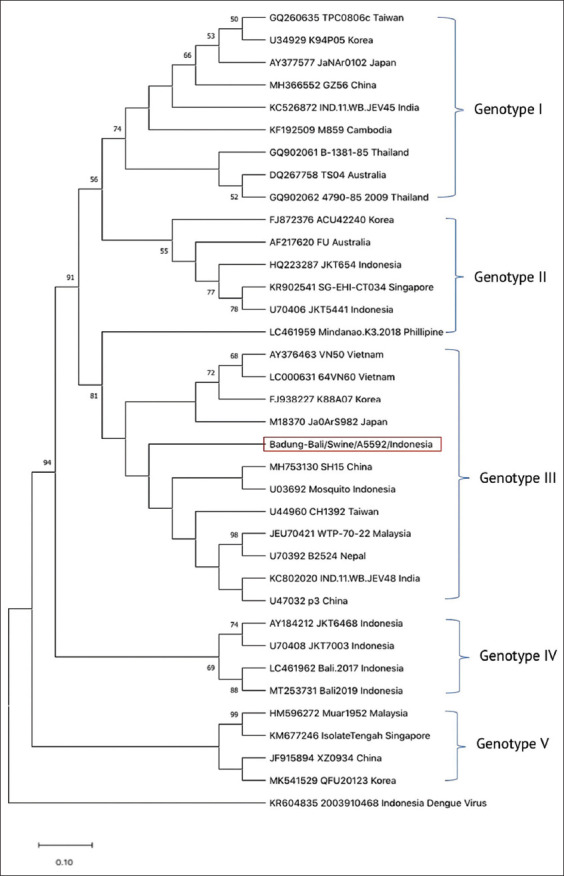
The evolutionary history was inferred using the maximum Likelihood method and Kimura 2-parameter model. The tree with the highest log likelihood (−1772.26) is shown. The percentage of trees in which the associated taxa clustered together is shown next to the branches. There were a total of 36 positions in the final dataset.

Sanger sequencing indicated that the product amplified from A5595 was due to non-specific amplification, and that only A5592 was specific for the envelope gene of JEV, as determined by blast search. We compared the nucleotide sequence of A5592 (length of the final sequence 252 bp) with 35 other representative sequences spanning all five genotypes from various countries. On the basis of the phylogenetic analysis, A5592 clustered within JEV genotype III (Figure-3).

### Mosquito identification

The trapped mosquitoes collected in Denpasar City and Badung Regency were similar in genus and species compositions. However, they were significantly different in total number of mosquitoes collected during the 6-month study period. Two genera of the mosquitoes trapped were *Anopheles* spp. and *Culex* spp. The *Anopheles* spp. were not further delineated; however, the trapped *Culex* spp. were identified further for their species as the primary vector of JEV in the endemic areas [12, 19].

**Figure-4:**
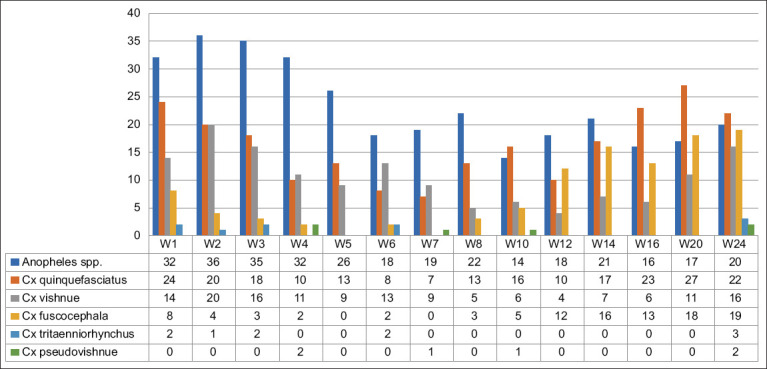
Number of mosquitoes trapped in a pig sentinel in Denpasar.

A total of 822 trapped mosquitoes collected in Denpasar were identified. These mosquitoes consisted of 326 *Anopheles* spp. and 496 *Culex* spp. Five species of the trapped *Culex* spp. were identified, consisting of *Culex quinquefasciatus* 228, *Culex vishnui* 147, *Culex fuscocephala* 105, *C. tritaeniorhynchus* 10, and *Culex pseudovishnui* 6 (Figure-4).

**Figure-5:**
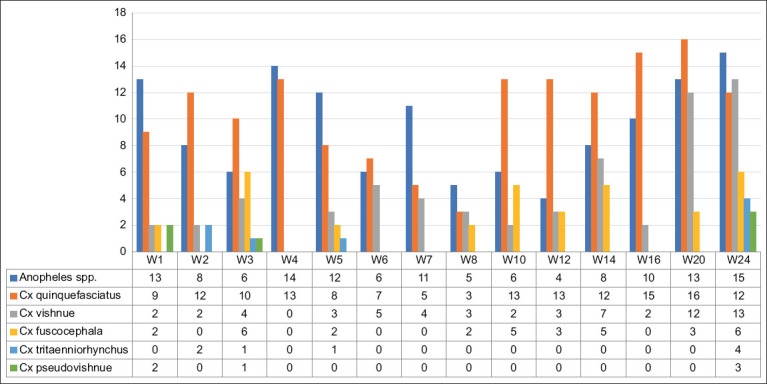
Number of mosquitoes trapped in a pig sentinel in Badung Regency.

Three hundred and eighty-nine of the trapped mosquitoes were collected at the pig sentinel site in Badung area. Of these, 131 were *Anopheles* spp. and 258 were *Culex* spp. identified as *C. quinquefasciatus* (148), *C. vishnui* (62), *C. fuscocephala* (34), *C. tritaeniorhynchus* (8), and *C. pseudovishnui* (6) (Figure-5).

## Discussion

Pigs play an important role in the circulation of JEV in the environment [20] and play a major role as amplifying hosts and sources of JEV [9]. Furthermore, pigs are effective sentinel animals for monitoring JEV transmission and circulation in the field [21, 22] through viral and serological detection.

It has been reported that viremia in JEV-infected pigs may be present for up to 4 days [16, 23], during which the mosquitoes can feed on the blood of infected pigs and transmit the virus through their bites into other susceptible hosts, including humans. Therefore, it is possible to estimate the period of viremia by determining the period of seroconversion in the animals. In consequence, it is also possible to predict the risk of the virus being fed on by the mosquito vectors and transmitted to other susceptible hosts, including epidemic JE cases in humans [22, 24, 25].

This study showed that maternal antibodies against JEV were likely present at varying levels in all piglets tested, consistent with the detection of antibodies in sera from all sows tested, providing immunity to newborn piglets in the 1^st^ weeks of life [26].

In agreement with the previous studies, these results suggest that maternal-derived antibodies (MDA) may exist at a detectable level in the early weeks of newborn weaning piglets [27]. MDA-immune piglets may be protected from infection in the 1^st^ weeks of their age from maternal antibodies in the colostrum [28]. As the MDA decays, the piglets become susceptible to JEV infection. In endemic areas, JEV infection is likely to occur after weaning the piglets [2]. On the basis of the OD results, however, seropositivity of the antibodies against JEV was erratically detected in all sample piglets tested from week 1 to week 8 before they remained stable in weeks 10–24 (sera of pigs D2 and D12).

The constant high OD values of the sera of the sampled piglets in detecting antibodies from the 1^st^ week of age made it difficult to estimate seroconversion. In this study, the IgG antibody against JEV was relatively stable in most of the serum samples from both pig sentinels in Denpasar and Badung during the study period. However, the classical pattern of JEV seroconversion was not observed in most of the tested samples. This may be due to the high intensity of JEV circulation and transmission in the area in which repeated JEV exposure might have occurred in the pigs during the study period because Bali is a JE hyperendemic area [13, 29]. This suggests that the sample pigs in both sentinel settings of peri-urban and urban areas in Bali were likely to have been infected with JEV before 6 months of age or when they reached sexual maturity. This study result agrees with the study conducted by Cappelle *et al*. [30], in which 96.6% (28/29) of the sentinel pigs in the peri-urban area of Phnom Penh, Cambodia, seroconverted before the age of 6 months.

Due to the difficulty in detecting the classical pattern of seroconversion in most of the samples, the median time of seroconversion could not be estimated. This may be due to weekly blood collection from the 1^st^ to 8^th^ week of the sample piglets’ age, followed by fortnightly blood collection until the pigs reached 16 weeks and every 4 weeks until the pigs were 24 weeks of age. In infected pigs, viremia caused by JEV has been reported to occur for up to 4 days, followed by the release of antibodies [31]. The period of seroconversion may have been missed, especially if it had changed to fortnightly or monthly bleeding after the end of the MDA. Alternatively, pigs may have been continuously exposed throughout the study; therefore, seroconversion was masked during the transition from maternal to induced antibodies. As a result, all of the tested sampled pig sera in this study were positive for antibodies, even though their titers were different.

A significant increase in antibody titer was observed in pig serum B9 collected from week 4 to week 8, illustrating the classical pattern of seroconversion. The serum antibody titer was four-fold higher in week 8 than in week 4, suggesting that natural JEV infection and viremia likely occurred in weeks 5–6. In addition, the genetic sequence of cluster JEV genotype III was detected in the pooled blood of sample pig B9 collected from week 5 to week 8 (A5592), indicating that infection occurred during this period. However, the limited seroconversion identified in the present study makes it difficult to infer the true time of infection. It may be possible to demonstrate seroconversion using a much larger sample size (which was not in the scope of this study), which is a potential focus for future studies. In addition, JEV antibody-free sampled piglets or pigs should be used in future studies to evaluate the dynamics of JEV antibody seroconversion.

The sampled piglets appeared to have natural JEV infection after weaning and/or at 6 months of age without any specific clinical signs and produced long-life antibodies. If immunity develops at an early age, there may be no adverse clinical signs at the time of re-infection, even after 6 months of age. In particular, there would be no reproductive disorders during re-infection at the time of sexual maturity due to neutralizing antibodies. Early JEV infection in pigs in Bali has also been predicted to occur before the age of 6 months [32], and it is commonly found in tropical endemic countries where high circulation of JEV occurs [30].

The present study also revealed the presence of JEV infection in pigs in urban and peri-urban areas of Bali Province, Indonesia, indicating that this infection is potentially as significant as that observed in rural areas [13]. As a result, control and monitoring programs against JEV infection should focus not only on rural areas but also on urban and peri-urban areas.

Although the commercial ELISA used in this study is reported to have high specificity (98.2%) (communication with Elabscience), the results may be somewhat clouded as other flaviviruses that generate cross-reactive antibodies, especially within the JE serogroup, are circulating in the province [9] and can result in nonspecific results. Plaque reduction neutralization testing (PRNT) is the gold standard for determining the specificity of flavivirus antibody responses; however, PRNT testing was unavailable for animal samples in the study area.

Phylogenetic analysis of the nucleotide sequence of B9 pig blood sample (sample ID A5592) collected from Badung showed that it belonged to JEV genotype III. This is consistent with JEV genotypes previously reported in Indonesia. In addition to genotype V, the other four JEV genotypes have been detected and reported [14]. JEV genotypes I and III infections tend to be more prevalent in JEV-infected humans than other genotypes [33], including cases in tropical areas of Southeast Asia [34]. Further studies are required to assess which JEV genotype is dominant and related to human cases in the Bali area.

The species composition of mosquitoes collected in Denpasar and Badung were similar. *C*. *tritaeniorhynchus* is the primary vector of JEV among *Culex* spp. mosquitoes [35]. *C. tritaeniorhynchus* was rarely detected in this study. This may be due to less attractive breeding sites in urban and peri-urban areas where traps were set compared to flooded areas, such as rice paddy fields or pond breeding sites commonly found in rural areas [36].

Although *Culex tritaeniorhynchus* is reported to be the primary vector of JEV, *Culex quinquefasciatus*, *Culex fuscocephala*, and *C. pseudovishnui* were also trapped in the present study. Some JEV mosquito vectors, such as 59.86% (1281/2140) of *C. tritaeniorhynchus*, 19.86% (425/2140) of *C. quinquefasciatus*, and 15.61% (334/2140) of *C. fuscocephala*, were also reported to be mainly trapped in small holder pig farms around Medan City in North Sumatra, another province in Indonesia [37], indicating that the mosquito vectors can be found in other areas of the country.

*C*. *quinquefasciatus* was predominant in all of the *Culex* mosquito samples collected in this study. These results suggest that the mosquito is likely to be more abundant in urban and peri-urban areas than other *Culex* spp. It is more attracted to breeding and laying its eggs in polluted water drainage, pits, and stagnant groundwater [5]. It has been reported that mosquitoes are also less frequently found in rural areas [9]. These results were similar to those of previous studies in similar environments where *C. quinquefasciatus* predominated in urban and peri-urban areas [30].

*C. quinquefasciatus* is a zoo-anthropophilic mosquito that can feed on both animals and humans [38]. This characteristic is crucial for the transmission of JEV from infected pigs to susceptible humans, especially for those who are closely associated with pigs and mosquitoes. In this situation, it appears that pig farmers and their family members, in particular those <15 years of age who live close to their pigs in JEV-endemic areas, are at high risk of exposure to the virus.

Moreover, in this study, mosquito light traps were set without bait, which makes them less attractive to mosquitoes. Carbon dioxide should be added to mosquito light traps when they are placed every day. In the absence of bait, it is likely that fewer mosquitoes were attracted to the light traps even if the UV light in the light trap was turned on during the collection period. Due to logistical constraints, it was not possible to source the carbon dioxide bait for this study.

Other limitations to be acknowledged in this study are that the study only used two different sentinel settings in Denpasar City and Badung Regency in Bali, which do not represent the ecology of Bali in general. In addition, the field study was performed over a period of 6 months and did not reflect the annual period of the season in the study area even though only dry and rainy seasons exist each year. This may also lead to undetected variation in JEV dynamics in relation to the annual climatic variable in the study area, which can be an important predictor in relation to the number of human JE cases. However, the 6-month study period estimates JEV activity with the seasonal period in the region. The first 3 months of the study, from September to November 2020, were the end of the dry season and the beginning of the rainy season in Bali, from December 2020 to February 2021.

## Conclusion

JEV seroconversion is likely to occur in pigs in Badung Regency before the pig reaches sexual maturity. Sequence data indicate that JEV genotype III is circulating in the pig sentinel setting in the regency; however, circulating genotypes need to be clarified through increased surveillance. Meanwhile, *Culex* spp., *C. quinquefasciatus*, and *Anopheles* spp. were the dominant mosquitoes trapped present in the study sites set in the urban area of Denpasar and peri-urban areas of Badung, Bali, which potentially have a role for being JEV vectors.

## Authors’ Contributions

IMK, SS, MB, and MO: Conceptualized and designed the study and reviewed and edited the manuscript. IMK, AAAMA, INMA, and IBMO: Set up the sentinel study, collected and tested the samples, and identified trapped mosquitoes. IMK, MB, and MO: Performing data analysis. All authors have read, reviewed, and approved the final version of the manuscript.

## Appendix

**Appendix 1:** The ELISA OD values of the 11 pig sera collected from week 1 to week 8 of the piglets’ age. First test result of ELISA kit on detecting seroconversion antibodies against JEV from sampled piglets in pig sentinel settings in Denpasar and Badung. The 1^st^ table contains the ELISA OD values, while the 2^nd^ table describes the tested pig sera of D2, D4, D6, D12, D14, B3, B7, B9, B11, B13, and B17 collected from week 1 to week 8.

Sample ID	1	2	3	4	5	6	7	8	9	10	11	12
A	2.049	2.132	1.576	1.993	1.594	1.243	0.685	2.185	1.723	1.281	0.975	2.855
B	1.4	1.576	1.871	1.751	2.193	1.102	0.897	2.232	1.872	0.927	0.871	2.99
C	1.912	1.745	1.67	1.037	1.638	0.912	1.019	1.348	1.077	1.45	0.798	0.255
D	2.356	1.804	1.805	1.263	1.762	1.305	0.892	0.921	1.048	0.695	0.953	0.082
E	1.922	1.808	1.809	2.114	1.471	1.199	1.043	1.1	1.015	0.82	0.988	2.613
F	1.807	1.672	1.863	2.003	1.593	1.099	0.745	0.96	0.958	0.924	0.89	1.877
G	2.025	1.748	1.899	2.115	1.399	1.383	0.667	1.062	1.359	1.118	1.795	2.297
H	1.999	1.937	2.264	1.773	0.494	1.495	1.266	1.377	1.308	0.848	1.82	0.722

**Weeks**	**1**	**2**	**3**	**4**	**5**	**6**	**7**	**8**	**9**	**10**	**11**	**12**

W1	D2	D4	D6	D12	D14	B3	B7	B9	B11	B13	B17	Positive control
W2	D2	D4	D6	D12	D14	B3	B7	B9	B11	B13	B17	Positive control
W3	D2	D4	D6	D12	D14	B3	B7	B9	B11	B13	B17	Negative control
W4	D2	D4	D6	D12	D14	B3	B7	B9	B11	B13	B17	Blank
W5	D2	D4	D6	D12	D14	B3	B7	B9	B11	B13	B17	Sow D1
W6	D2	D4	D6	D12	D14	B3	B7	B9	B11	B13	B17	Sow D2
W7	D2	D4	D6	D12	D14	B3	B7	B9	B11	B13	B17	Sow B1
W8	D2	D4	D6	D12	D14	B3	B7	B9	B11	B13	B17	Sow B2
